# In situ follicular neoplasm discovered as an enlarging pulmonary nodule complicated by pulmonary aspergillosis

**DOI:** 10.1111/1759-7714.14878

**Published:** 2023-04-03

**Authors:** Tsuyoshi Uchida, Hirochika Matsubara, Ryunosuke Koizumi, Harunobu Sasanuma, Aya Sugimura, Yuichiro Onuki, Hiroyuki Nakajima, Naoki Oishi

**Affiliations:** ^1^ University of Yamanashi Faculty of Medicine, General thoracic surgery Shimokato1110 Chuo Yamanashi Japan; ^2^ Department of Pathology University of Yamanashi Faculty of Medicine, University of Yamanashi Chuo Yamanashi Japan

**Keywords:** follicular lymphoma, lymph nodes, solitary pulmonary nodule

## Abstract

In situ follicular B cell neoplasm, previously known as follicular lymphoma in situ, is a neoplastic proliferation of follicular lymphoma‐like B cells confined to the germinal centers. Herein, we report a case of a woman in her 70s who initially presented with several enlarged abdominal lymph nodes. Seven months later during follow‐up, a solitary pulmonary nodule was detected. As it was close to the hilum, lobectomy was performed. The intraoperative frozen section showed fibrosis and a collection of lymphocytes and macrophages. Therefore, the lymph nodes were sampled. Station 4 and 10 lymph nodes exhibited similar tumor cells and were immunohistochemically positive for CD10 and BCL2. Thus, the patient was diagnosed with in situ follicular neoplasm and is currently under observation. In situ follicular neoplasm is typically a slowly progressive neoplasm; however, it can present as a rapidly enlarging pulmonary nodule complicated by pulmonary aspergillosis.

## Graphical abstract

We report a case of a woman in her 70s who initially presented with several enlarged abdominal lymph nodes. Seven months later during follow‐up, a solitary pulmonary nodule was detected. As it was close to the hilum, lobectomy was performed. Tumor cells of the specimen were immunohistochemically positive for CD10 and BCL2. Thus, the patient was diagnosed with in situ follicular neoplasm and is currently under observation. In situ follicular neoplasm is typically a slowly progressive neoplasm; however, it can present as a rapidly enlarging pulmonary nodule complicated by pulmonary aspergillosis.

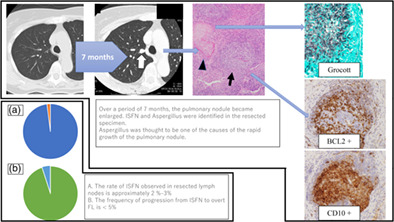

## INTRODUCTION

In situ follicular neoplasia (ISFN) is a neoplastic proliferation of follicular lymphoma‐like B cells confined to the germinal centers. Occasionally, it is detected as an incidental finding following lymph node resection for another lesion.^1^ The previously described follicular lymphoma (FL) in situ has now been reclassified as ISFN according to the 2017 World Health Organization classification.^1^ ISFN diagnosis based on imaging information alone is difficult, even though it is described as a benign tumor.

Herein, we report a case of ISFN in a patient who underwent pulmonary resection for a pulmonary nodule for which no definitive diagnosis had been made.

## CASE REPORT

**FIGURE 1:**
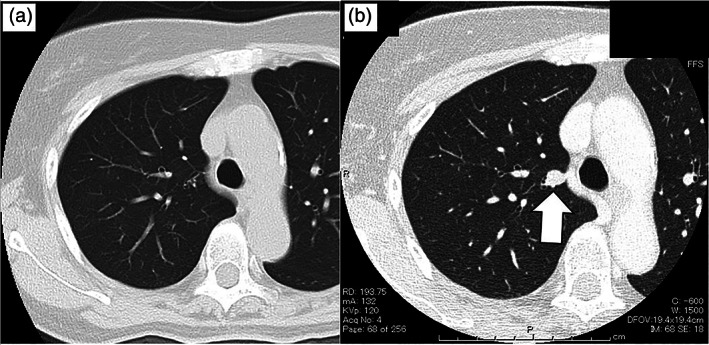
(a) Chest computed tomography (CT) image 4 months after the initial examination revealed no apparent nodule. (b) CT findings 7 months later (a) revealed a 1 cm diameter pulmonary nodule on the right side of the azygos vein (white arrow).

A woman in her 70s with a suspected pancreatic cyst was referred to the gastroenterology department at our institute. Abdominal ultrasound revealed no pancreatic cyst; however, several enlarged intra‐abdominal lymph nodes with nonspecific inflammatory changes were visualized. Chest computed tomography (CT) performed 4 months later did not show any apparent nodules; however, a repeat CT performed 7 months after detected a 1 cm lung nodule (Figure 1). The nodule had a standardized uptake value of 2.24 on 18F‐fluorodeoxyglucose positron emission tomographic (PET) imaging. There were no other nodules with abnormal standardized uptake. The patient was referred to our department for surgical biopsy and resection. The patient's general condition was unremarkable; however, the renal function was mildly disturbed (creatinine, 0.82 g/dL; estimated glomerular filtration rate, 51 mL/min). Thoracoscopic upper lobectomy of the right lung was performed. A needle biopsy or partial resection should have been performed to confirm malignancy by rapid pathology, followed by anatomical lobectomy; however, both methods were deemed difficult because of the proximity of the nodule to the pulmonary hilum; thus, lobectomy was performed with four‐port complete thoracoscopic surgery and was completed with no complications.

**FIGURE 2:**
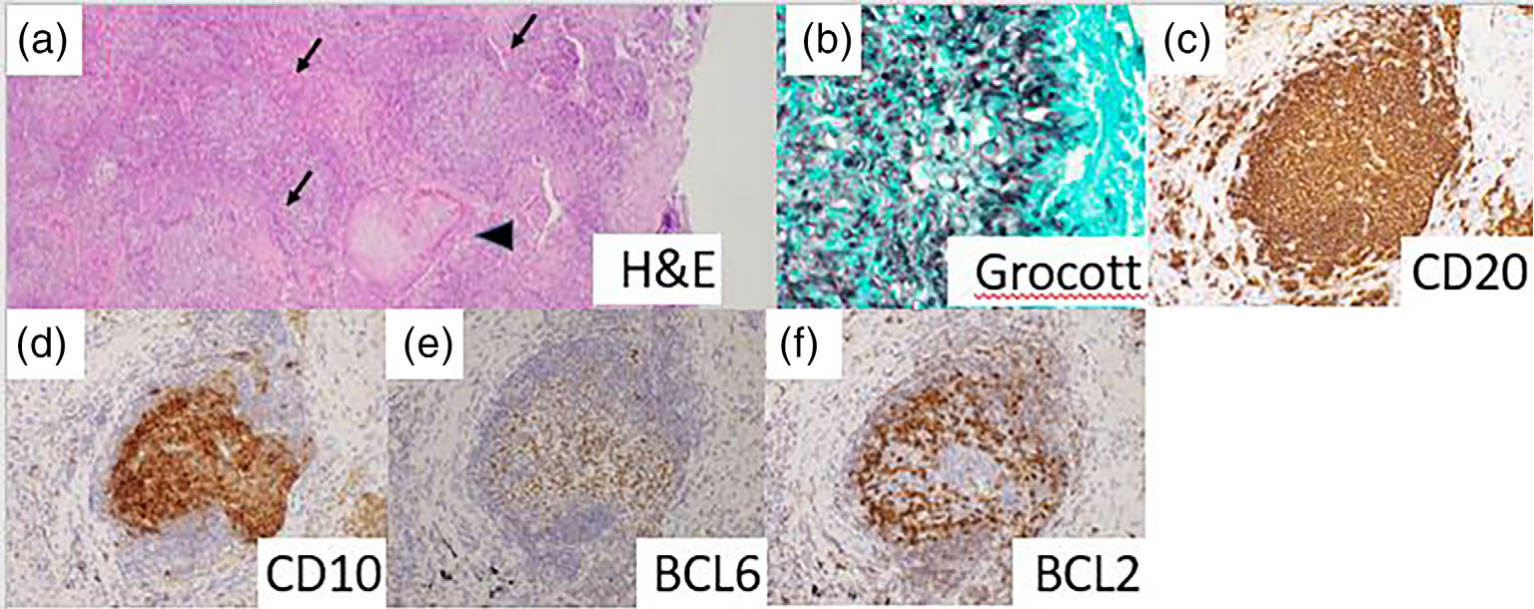
(a) Hematoxylin and eosin staining shows reactive‐like follicles (black arrow) and aspergillus colonization (black triangle). (b) Grocott staining shows aspergillus bodies. (c) CD20 was positive on the pulmonary nodule and lymph nodes. (d) CD10 was positive on the pulmonary nodule and lymph nodes. (e) BCL6 was positive on the pulmonary nodule and lymph nodes.

The intraoperative frozen tumor section showed only fibrosis and lymphocyte and alveolar macrophage collections instead of malignant changes; thus, the lymph nodes were sampled. The postoperative course was uneventful. The results of the pathological examination revealed an Aspergillus‐like fungal mass in the center of the tumor. The surrounding area was infiltrated by lymphocytes forming numerous lymphoid follicles. Based on several immunostaining results, ISFN, previously known as in situ FL, was diagnosed (Figure 2). Both station 10 and 4 lymph nodes had similar tumor cells, and station 4 lymph nodes showed a disorganized distribution of germinal centers in the tumor. The patient has currently been under postoperative observation for 30 months.

## DISCUSSION

This case demonstrates two important points. First, in situ follicular neoplasm can manifest as a rapidly enlarging pulmonary nodule. In the present case, enlarged lymph nodes were already observed in the abdominal cavity; however, they did not change significantly during the disease. The pulmonary nodule was observed on the follow‐up CT. The interval between the first CT scan when no nodules were observed, and the second CT scan when they were observed, was 3 months, indicating a fast growth rate. ISFN is described as a slow‐progressing tumor, but the pulmonary nodule in the present case had an extremely fast growth rate. When pulmonary nodules rapidly increase in size, as in this case, the differential diagnosis is infection or malignancy rather than a benign tumor or ISFN. Moreover, in the current case, the pathological examination revealed an Aspergillus‐like fungal mass in the resected specimen. Therefore, pulmonary aspergillosis should be included in the differential diagnosis of the enlarged pulmonary nodules, which could explain the absence of enlarged intra‐abdominal lymph nodes. Furthermore, if ISFN had been considered in the differential diagnosis, a definite diagnosis would require resection.^1^

ISFN does not require aggressive treatment; however, careful follow‐up is needed. The rate of ISFN observed in resected lymph nodes is approximately 2%–3%, and the frequency of progression to overt FL is <5%.^2^, ^3^, ^4^, ^5^ In this case, the preoperative PET did not indicate metastasis of ISFN; however, it was observed both in the pulmonary nodule and sampled lymph nodes. This indicates that ISFN cells can metastasize lymphatically throughout the body. Cells may be found in the blood and lymph nodes throughout the body in ISFN cases.^5^ Metastasis implies that the disease will recur or increase in size, as in thymoma, and it may even transform into FL. The accurate differentiation between the ISFN and FN is challenging but of great importance to avoid overtreatment^6^; therefore, careful follow‐up is necessary.

## AUTHOR CONTRIBUTIONS

Conceptualization, Tsuyoshi Uchida and Ryunosuke Koizumi; Methodology, Tsuyoshi Uchida; Investigation, Naoki Oishi, Ryunosuke Koizumi and Harunobu Sasanuma; Formal Analysis, Tsuyoshi Uchida and Ryunosuke Koizumi; Resources, Tsuyoshi Uchida and Ryunosuke Koizumi; Writing ‐ Original Draft, Tsuyoshi Uchida and Yuichiro Onuki; Writing ‐ Review & Editing, Hirochika Matsubara and Aya Sugimura; Supervision, Hiroyuki Nakajima.

## CONFLICT OF INTEREST STATEMENT

The authors declare that they have no conflicts of interest.

## References

[1] Tamber GS , Chévarie‐Davis M , Warner M , Séguin C , Caron C , Michel RP . In‐situ follicular neoplasia: a clinicopathological spectrum. Histopathology. 2021;79:1072–86. 10.1111/his.14535 34333806

[2] Henopp T , Quintanilla‐Martínez L , Fend F , Adam P . Prevalence of follicular lymphoma in situ in consecutively analysed reactive lymph nodes. Histopathology. 2011;59:139–42. 10.1111/j.1365-2559.2011.03897.x 21771030

[3] Bermudez G , González de Villambrosía S , Martínez‐López A , et al. Incidental and isolated follicular lymphoma in situ and mantle cell lymphoma in situ lack clinical significance. Am J Surg Pathol. 2016;40:943–9. 10.1097/PAS.0000000000000628 26945339

[4] Jegalian AG , Eberle FC , Pack SD , Mirvis M , Raffeld M , Pittaluga S , et al. Follicular lymphoma in situ: clinical implications and comparisons with partial involvement by follicular lymphoma. Blood. 2011;118:2976–84. 10.1182/blood-2011-05-355255 21768298 PMC3175777

[5] Oishi N , Segawa T , Miyake K , Mochizuki K , Kondo T . Incidence, clinicopathological features and genetics of in‐situ follicular neoplasia: a comprehensive screening study in a Japanese cohort. Histopathology. 2022;80:820–6. 10.1053/j.semdp.2017.11.001 35038193

[6] Oishi N , Montes‐Moreno S , Feldman AL . In situ neoplasia in lymph node pathology. Semin Diagn Pathol. 2018;35:76–83. 10.1111/his.14617 29129357

